# Low-Concentration Arsenic Trioxide Inhibits Skeletal Myoblast Cell Proliferation via a Reactive Oxygen Species-Independent Pathway

**DOI:** 10.1371/journal.pone.0137907

**Published:** 2015-09-11

**Authors:** Shing Hwa Liu, Rong-Sen Yang, Yuan-Peng Yen, Chen-Yuan Chiu, Keh-Sung Tsai, Kuo-Cheng Lan

**Affiliations:** 1 Institute of Toxicology, College of Medicine, National Taiwan University, Taipei, Taiwan; 2 Department of Medical Research, China Medical University Hospital, China Medical University, Taichung, Taiwan; 3 Departments of Orthopaedic, College of Medicine and Hospital, National Taiwan University, Taipei, Taiwan; 4 Departments of Laboratory Medicine, College of Medicine and Hospital, National Taiwan University, Taipei, Taiwan; 5 Department of Emergency Medicine, Tri-Service General Hospital, National Defense Medical Center, Taipei, Taiwan; INSERM-Université Paris-Sud, FRANCE

## Abstract

Myoblast proliferation and differentiation are essential for skeletal muscle regeneration. Myoblast proliferation is a critical step in the growth and maintenance of skeletal muscle. The precise action of inorganic arsenic on myoblast growth has not been investigated. Here, we investigated the *in vitro* effect of inorganic arsenic trioxide (As_2_O_3_) on the growth of C2C12 myoblasts. As_2_O_3_ decreased myoblast growth at submicromolar concentrations (0.25–1 μM) after 72 h of treatment. Submicromolar concentrations of As_2_O_3_ did not induce the myoblast apoptosis. Low-concentration As_2_O_3_ (0.5 and 1 μM) significantly suppressed the myoblast cell proliferative activity, which was accompanied by a small proportion of bromodeoxyuridine (BrdU) incorporation and decreased proliferating cell nuclear antigen (PCNA) protein expression. As_2_O_3_ (0.5 and 1 μM) increased the intracellular arsenic content but did not affect the reactive oxygen species (ROS) levels in the myoblasts. Cell cycle analysis indicated that low-concentrations of As_2_O_3_ inhibited cell proliferation via cell cycle arrest in the G1 and G2/M phases. As_2_O_3_ also decreased the protein expressions of cyclin D1, cyclin E, cyclin B1, cyclin-dependent kinase (CDK) 2, and CDK4, but did not affect the protein expressions of p21 and p27. Furthermore, As_2_O_3_ inhibited the phosphorylation of Akt. Insulin-like growth factor-1 significantly reversed the inhibitory effect of As_2_O_3_ on Akt phosphorylation and cell proliferation in the myoblasts. These results suggest that submicromolar concentrations of As_2_O_3_ alter cell cycle progression and reduce myoblast proliferation, at least in part, through a ROS-independent Akt inhibition pathway.

## Introduction

Inorganic arsenic is well-known toxicant and a potent human carcinogen [1,2]. Chronic exposure to arsenic through consumption of polluted water is a major risk to the world population. Nevertheless, arsenic has also been used for many centuries as medicinal agents for the treatment of syphilis, ulcers, and psoriasis [3,4]. In addition, the U.S. Food and Drug Administration approved arsenic trioxide as a chemotherapeutic agent for treating acute promyelocytic leukemia (APL) [5]. Arsenic is also a transplacental carcinogen in rodents and probably in human [6,7]. Furthermore, the evidence demonstrates that arsenite induces human neuroblastoma cell proliferation via a vascular endothelial growth factor (VEGF) signaling pathway [8].

Arsenic is capable of producing reactive oxygen species (ROS), eliciting DNA damage and slowing cell-cycle progression [9]. Several studies have indicated that arsenic trioxide induces cell cycle arrest and apoptosis in various cells. These cells include lymphoid neoplasms [10] and the head and neck cancer cell line PCI-1 [11], human fibroblasts [12], human bronchial epithelial cell line BEAS-2B [13], and human colonic, breast, and pancreatic cancer cells [14]. The effect of inorganic arsenic on the cell cycle progression in myoblasts is still unclear.

In postnatal skeletal muscle, satellite cells (quiescent muscle precursor cells) reside beneath the basal lamina. They are the primary cellular source of muscle growth and regeneration [15,16]. In response to muscle injury, satellite cells activate, proliferate, and differentiate to form a pool of myoblasts, and then fuse together to repair or replace damaged muscle fibers [17]. In skeletal muscles undergoing hypertrophy, an increase in myonuclear numbers, which produced by the satellite cells, precedes the enlargement of myofiber size [18]. Evidence shows that maternal undernutrition during gestation results in a decrease in myoblast proliferation coupled with an earlier onset of differentiation to fibers. This alters the development of muscle fibers and reduces the birth weight of newborn lambs [19,20]. Therefore, myoblast proliferation is an early and critical cellular event essential for skeletal muscle growth.

Previous studies indicate that arsenic suppresses the myoblast differentiation (myogenesis) [21,22]. Arsenic also inhibits the osteoblast differentiation from bone marrow stromal cells [23]. Moreover, arsenic displays striking suppression of muscle regeneration in a mouse skeletal muscle injury model [22]. In addition, arsenic at micromolar concentrations induces apoptosis in the myoblasts [24]. However, the precise action of inorganic arsenic on myoblast proliferation remains to be clarified. In this study, we focused on the investigation of dose-related arsenic effects and possible mechanisms of action of inorganic arsenic (As_2_O_3_) on myoblast proliferation.

## Materials and Methods

### Cell culture

C2C12 mouse myoblasts were obtained from American Type Culture Collection (CRL-1772; Manassas, VA, USA) and cultured in growth medium consisting of Dulbecco’s modified Eagle’s medium (DMEM) supplemented with 10% fetal bovine serum (FBS) and antibiotics (100 U/ml penicillin, 100 μg/ml streptomycin). The cells were incubated in a 5% CO_2_ environment at 37°C.

As_2_O_3_ (Sigma-Aldrich, St. Louis, MO, USA) 0.1 M (stock solution) was prepared in 1 M NaOH, diluted to 10^−3^ M in PBS, and adjusted to pH 7.2 using HCl. It was diluted further with phosphate-buffered saline (PBS), and the solutions were kept at 4°C until use.

### Cell proliferation assay

Cell proliferation assays were conducted in six-well culture dishes. C2C12 myoblast cultures were seeded at 1×10^4^ cells per well in DMEM. After a 16-h attachment period, arsenic test media was added, which consisted of 10% fetal bovine serum media with 0–1 μM As_2_O_3_. Plates were then incubated in an atmosphere of 37°C and 5% CO_2_ for a further 24, 48 and 72 h. After the appropriate treatment period, cells were trypsinized, re-suspended in 0.4% trypan blue solution and counted using a hemocytometer. The positions of the samples on the plate were randomly assigned, and all samples were run in triplicate. Results are representative of at least three independent experiments.

### Bromodeoxyuridine incorporation assay

C2C12 myoblasts were cultured in growth medium with or without As_2_O_3_ (0.25, 0.5, and 1 μM) in 96-well microplates for 48 h. Subsequently, bromodeoxyuridine (BrdU) (Roche Diagnostics, Indianapolis, IN, USA) was added and the cells were incubated for an additional 4 h. After the culture supernatant was removed, the cells were fixed, and then incubated with an anti-BrdU antibody conjugated to peroxidase (anti-BrdU-POD). Bound anti-BrdU-POD was detected by a substrate reaction, and then quantified in an enzyme-linked immunosorbent assay (ELISA) plate reader.

### Annexin V-FITC apoptosis detection

Apoptosis was detected using an annexin V-fluorescein isothiocyanate (FITC) kit purchased from Becton-Dickinson (Franklin Lakes, NJ, USA). The assay was performed following the manufacturer’s instructions. Briefly, C2C12 cells were seed in 60 mm plates and cultured under the same condition as in the cell proliferation assay. Next, the cells were treated with As_2_O_3_ (0, 0.25, 0.5, and 1 μM) for 72 h. Both floating and attached cells were harvested and wash twice with ice-cold PBS. The cells were then re-suspended in 100 μl of binding buffer and incubated with annexin V-FITC and propidium iodide. After incubation for 30 min in the dark, 400 ml binding buffer was added to each tube and the samples were immediately analyzed using a FACScan flow cytometer (Becton-Dickinson).

### Cell cycle analysis

C2C12 cells were plated 2×10^4^ cells/100-mm-diameter tissue culture dishes without or with the indicated concentrations of As_2_O_3_ for 72 h. The cells were washed with PBS, detached with trypsin and fixed with 75% ethanol overnight. Samples were washed and re-suspended in 0.5 ml of PBS containing with 100 μg/ml RNase A and 5 μg/ml propidium iodide for 30 min. The DNA contents were measured by a FACScan flow cytometer (Becton-Dickinson). Ten thousand individual cells in each group were sampled.

### Protein extraction and immunoblotting

Cell lysates were prepared using radio-Immunoprecipitation assay (RIPA) buffer [10 mM Tris (pH 7.4), 150 mM NaCl, 1 mM ethylene glycol tetraacetic acid, 0.1% sodium dodecyl sulfate, 1 mM NaF, 1 mM sodium orthovanadate, 1 mM phenylmethylsulfonyl fluoride, 1 μg/mL aprotinin, and 1 μg/mL leupeptin]. The cell suspension was left on ice for 20 min, and then centrifuged at 10,000 × *g* for 20 min at 4°C. In some experiments, the nnuclear proteins were extracted using a nuclear extraction kit (Affymetrix, Fremont, CA, USA). Equal amounts of protein were subjected to sodium dodecyl sulfate-polyacrylamide gel electrophoresis (SDS-PAGE). Following electrophoresis, the proteins were electrotransferred to polyvinylidene difluoride membrane (Millipore, Billerica, MA, USA). The membranes were then blocked with 5% nonfat powdered milk for 1 h and subsequently incubated overnight at 4°C with primary antibodies [anti-p21, anti-p27, anti-cyclin-dependent kinase (CDK)2, anti-CDK4, anti-cyclin B1, anti-cyclin D1, anti-cell division cycle 2 (CDC2), anti-proliferating cell nuclear antigen (PCNA), anti-histone H1, anti-Akt1/2/3, anti-pERK, anti-pERK1, anti-α-tubulin (Santa Cruz Biotechnology, Santa Cruz, CA, USA) and anti-pAkt (Ser473) (Epitomics, Burlingame, CA)]. The membranes were next washed three times in Tris buffered saline and tween 20 (TBST) and incubated with horseradish peroxidase-conjugated goat anti-rabbit or anti-mouse immunoglobulin G (Millipore). The blots were developed using an enhanced chemiluminescence reagent and exposed to and X-ray film.

### Detection of arsenic contents

C2C12 myoblasts were treated with As_2_O_3_ (0.5–10 μM) for 24 h. After incubation, cells were harvested and washed with PBS three times followed by the addition of 0.1% nitric acid. The mixture was then votexed and frozen at -20°C overnight. The arsenic levels were determined in the supernatant containing intracellular arsenic by inductively coupled plasma mass spectrometry (ICP-MS). The detection limit for arsenic was approximately 0.1 ppb (μg/L).

### Measurement of intracellular ROS formation

A fluorescein-labeled dye, 2`,7`- dichlorofluorescein diacetate (DCFH-DA) was used to determine the generation of intracellular ROS. The non-fluorescent dye in cells was hydrolyzed to 2`,7`- dichlorofluorescein (DCF) upon interaction with intracellular ROS. Cells were incubated with 20 μM DCFH-DA for 30 min at 37°C, and then cells were washed twice with ice-cold PBS and harvested. The cells were immediately analyzed using a FACScan flow cytometer (Becton Dickinson) to determine the ROS generation.

### Statistical analysis

Results were expressed as means ± SEM. The significant differences from the respective controls for each experimental test condition were assessed by analysis of variance (ANOVA) and the Bonferroni t-test with *P* < 0.05 considered significant.

## Results

### Effects of submicromolar As_2_O_3_ on myoblast growth and apoptosis

**Fig 1:**
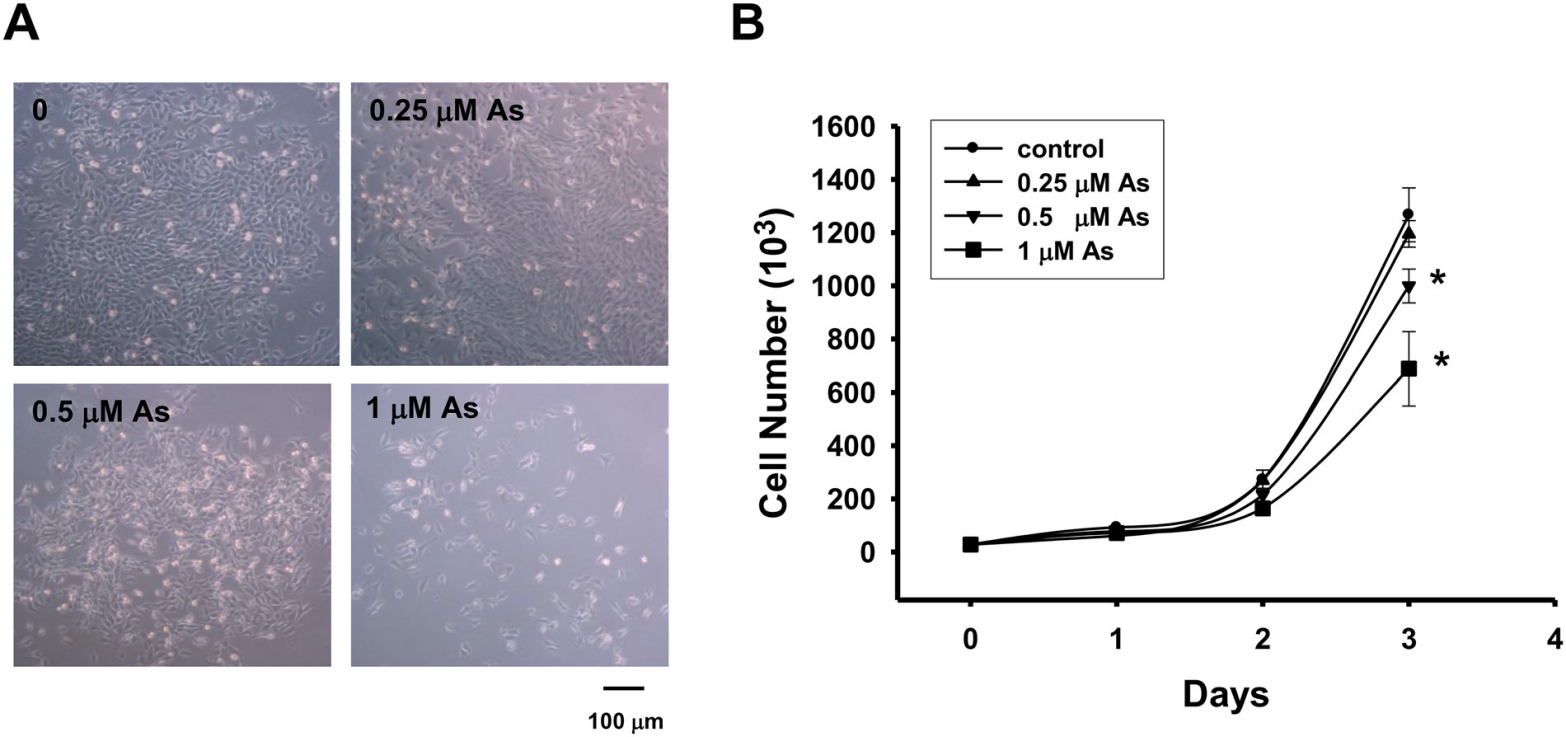
As_2_O_3_ decreases myoblast growth. C2C12 myoblasts were cultured with or without As_2_O_3_ (0.25, 0.5 and 1 μM) for 24–72 h. (A) Cell morphology in culture was assessed by light microscopy after incubation for 72 h in the presence or absence of As_2_O_3_. (B) The cell numbers were determined using trypan blue assay. Data are presented as means ± SEM of three independent experiments. *P < 0.05 vs control.

To determine the effect of As_2_O_3_ on the cell growth of myoblasts, the C2C12 cells were cultured in growth media with or without the treatment of As_2_O_3_ (0.25–1 μM) for 24–72 h. As shown in Fig 1, As_2_O_3_ reduced the C2C12 myoblast growth in a dose and time-dependent manner. Treatment with 0.5 and 1 μM As_2_O_3_ for 72 h significantly decreased C2C12 myoblast growth.

**Fig 2:**
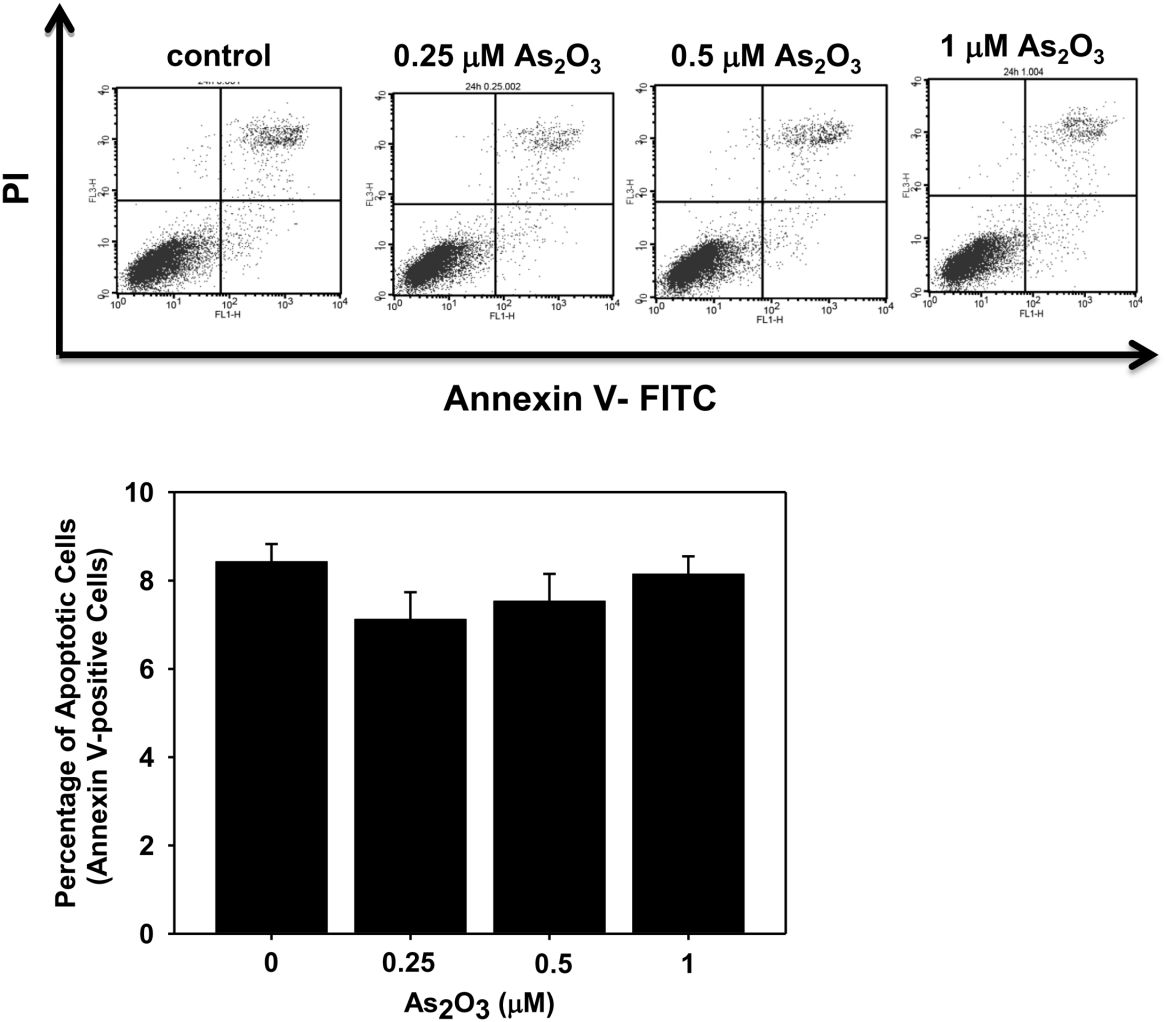
Low-concentration As_2_O_3_ does not induce myoblast apoptosis. (A) C2C12 myoblasts were cultured with or without As_2_O_3_ (0.25, 0.5 and 1 μM) for 72 h. Apoptotic cells were identified using an annexin-V assay. The percentage of annexin-V positive cells was calculated. Data are presented as means ± SEM of three independent experiments. No significant differentiations were observed between the groups (*P* > 0.05).

We next investigated whether or not apoptosis was involved in the myoblast growth inhibition by As_2_O_3_. As shown in Fig 2, As_2_O_3_ (0.25–1 μM) did not induce the myoblast apoptosis determined by annexin-V staining after 72 h treatment.

### Effects of low-concentration As_2_O_3_ on PCNA protein expression and BrdU incorporation

**Fig 3:**
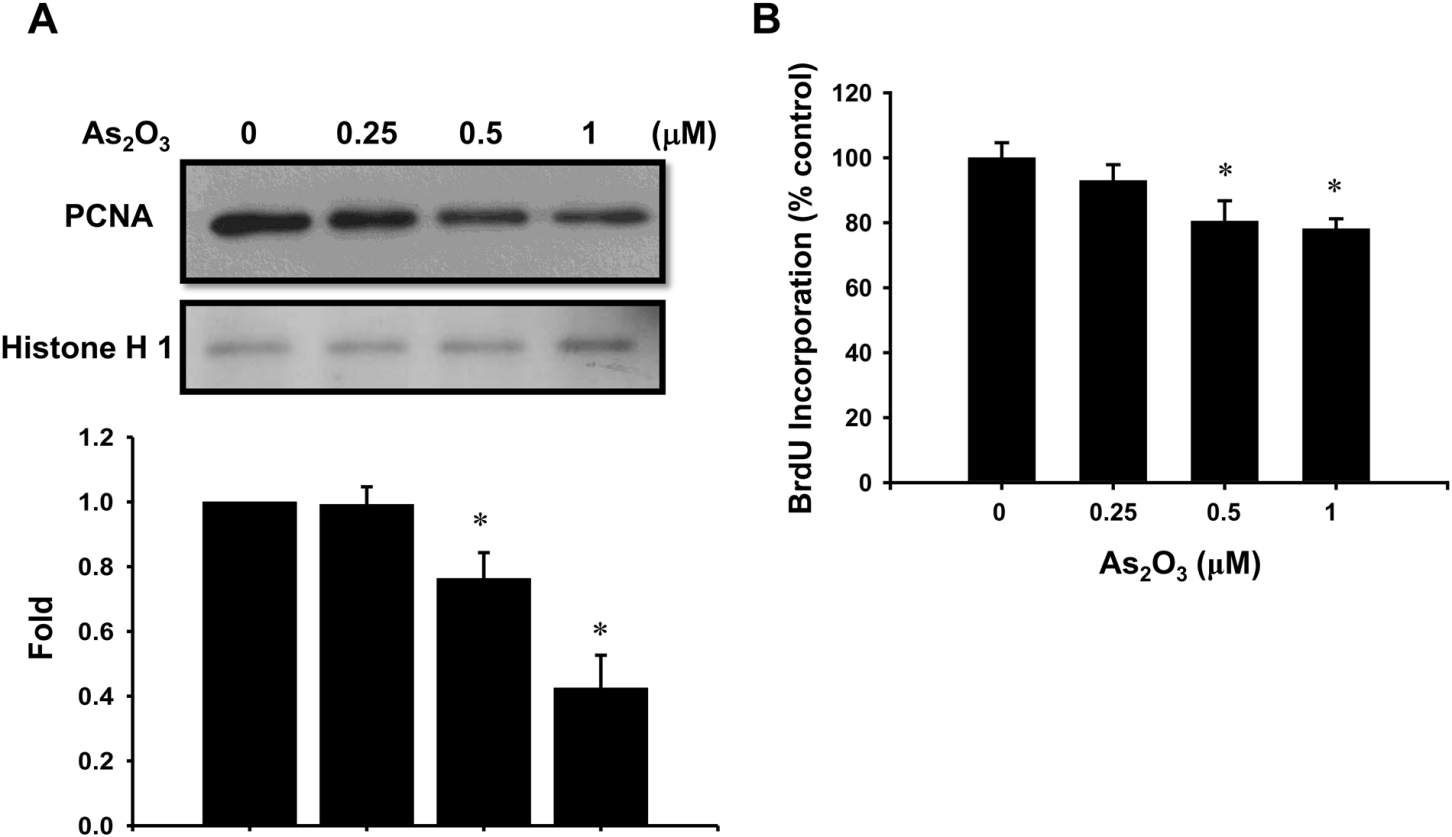
As_2_O_3_ decreases the nuclear levels of proliferating cell nuclear antigen (PCNA) protein expression and bromodeoxyuridine (BrdU) incorporation into the myoblasts. (A) C2C12 myoblasts were treated with As_2_O_3_ (0.25–1 μM) for 72 h. After incubation, the nuclear extracts were prepared from the cells and analyzed by Western blotting. Histone-1 expression was used as the internal control. PCNA protein expression was quantified by densitometry. (B) C2C12 myoblasts were treated with As_2_O_3_ (0.25–1 μM) for 48 h. BrdU incorporation was measured using an ELISA kit. Data are presented as means ± SEM of three independent experiments. *P < 0.05 vs control.

Next, we confirmed the effect of low-concentration As_2_O_3_ (0.25–1 μM) on myoblast proliferation. As shown in Fig 3A, the nuclear protein expression of PCNA (a marker for cell proliferation) [25] in C2C12 myoblasts was significantly decreased in a dose-dependent manner after As_2_O_3_ treatment. Moreover, BrdU incorporation into the myoblasts, which reflects the rate of DNA synthesis, also significantly reduced in a dose-dependent manner after As_2_O_3_ treatment (Fig 3B). These results indicate that As_2_O_3_ inhibits the myoblast proliferation at submicromolar concentrations.

### Effects of low-concentration As_2_O_3_ on intracellular arsenic contents and ROS levels

**Fig 4:**
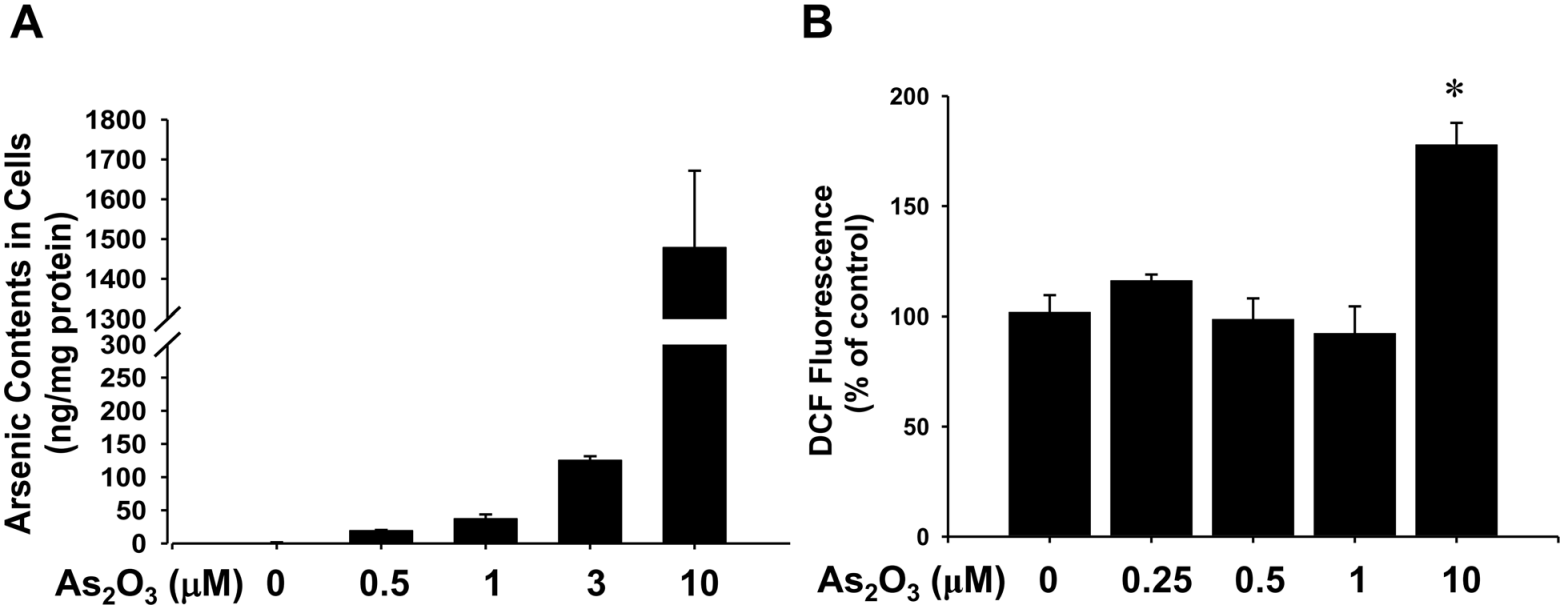
Detection of intracellular arsenic and reactive oxygen species (ROS) in myoblasts. C2C12 myoblasts were treated with As_2_O_3_ (0.5–10 μM) for 24 h (A) or 4 h (B). Arsenic contents and ROS levels in the cells were determined as described in Materials and Methods. Data are presented as means ± SEM for at least triplicate determinations. *P < 0.05 vs control.

ROS regulates high-concentration As_2_O_3_ (10–80 μM)-induced cell apoptosis [24,26]. Thus, we tested the arsenic contents and ROS levels in As_2_O_3_-treated myoblasts. As shown in Fig 4A, the arsenic contents of C2C12 myoblasts increased in a dose-dependent manner 24 h after treatment with As_2_O_3_ (0.5–10 μM). The generation of ROS in the myoblasts was determined by DCF fluorescence. As shown in Fig 4B, As_2_O_3_ 0.25–1 μM did not affect ROS generation in the myoblasts, but 10 μM of As_2_O_3_ markedly increased the intracellular ROS level.

### Effects of low-concentration As_2_O_3_ on G1 and G2-M phase arrest and protein expression of cell cycle-regulatory proteins

**Fig 5:**
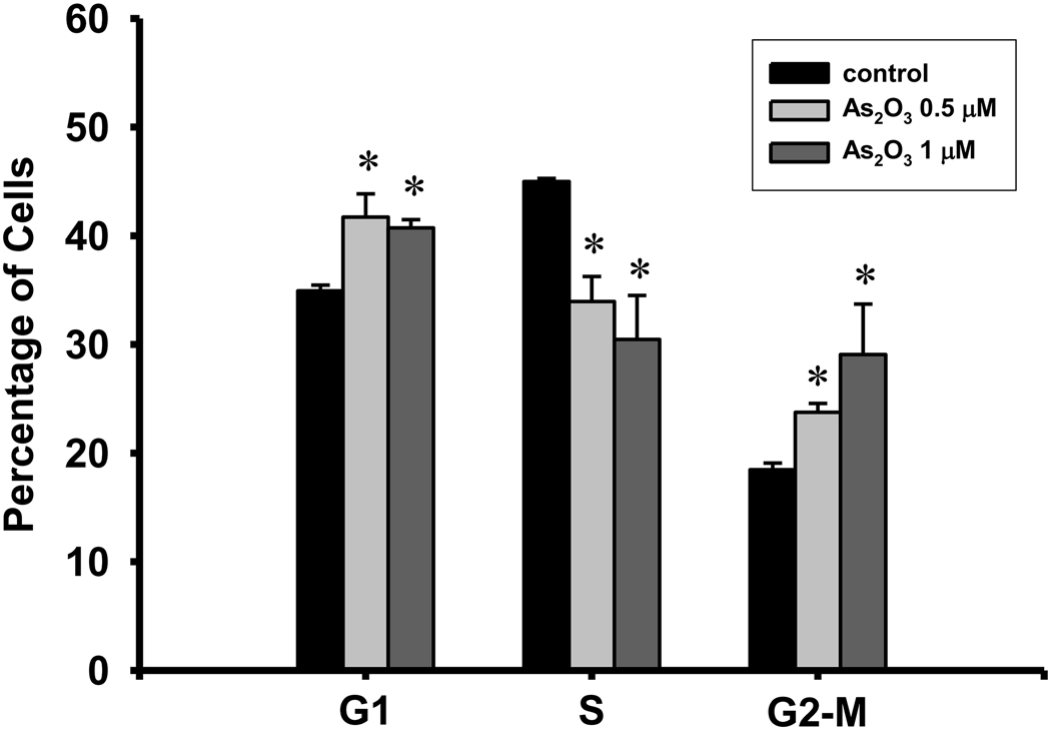
As_2_O_3_ alters the cell cycle phase distribution in myoblasts. C2C12 myoblasts were treated with As_2_O_3_ (0.5 and 1 μM) for 72 h. Cells were washed, fixed, and stained with PI, and then the DNA histogram was analyzed by flow cytometry. Data are presented as means ± SEM of three independent experiments. *P < 0.05 vs control.

We investigated whether or not low-concentrations of As_2_O_3_ affected the cell cycle distribution by flow cytometry. As shown in Fig 5, As_2_O_3_ (0.5 and 1 μM) significantly increased the percentages of C2C12 myoblasts in the G1 and G2/M phases after 72 h of exposure. This was accompanied by a significant decrease in the proportion of cells in the S phase. These results indicate that low-concentration inorganic arsenic inhibits the proliferation of C2C12 myoblasts by inducing G1 and G2/M phase cell cycle arrest.

**Fig 6:**
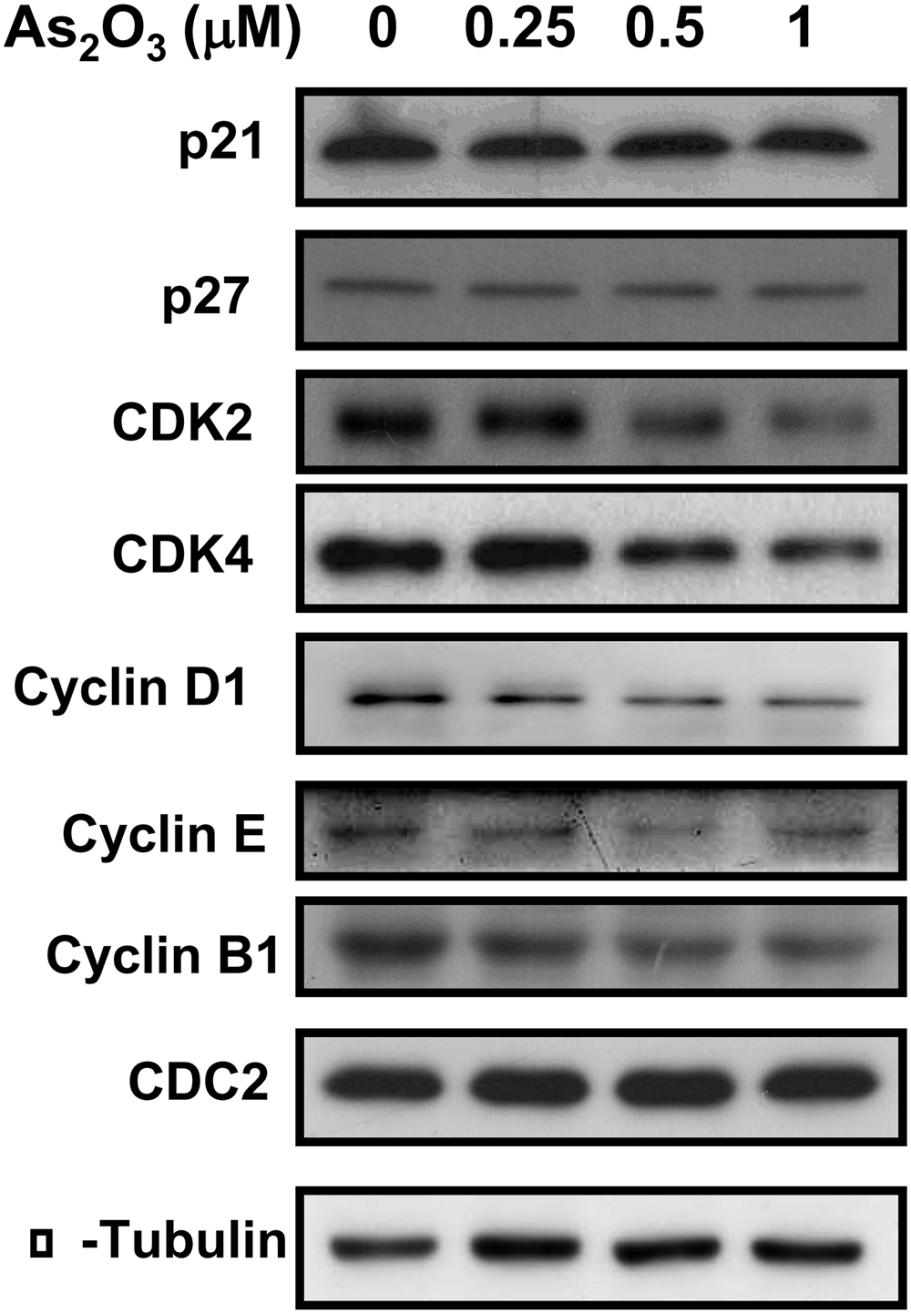
As_2_O_3_ affects the expressions of cell cycle-related proteins in myoblasts. C2C12 myoblasts were harvested at 48 h after incubation with As_2_O_3_ (0.25–1 μM). The protein expressions of p21, p27, cyclin-dependent kinase (CDK)2, CDK4, cyclin D1, cyclin E, cyclin B, and cell division cycle (CDC)2 were analyzed by Western blotting. Alpha-tubulin served as the sample equal loading control. Results are representative at least three independent experiments.

The cell cycle regulatory proteins were evaluated to determine the possible mechanism of low-concentration As_2_O_3_-altered cell cycle progression. As shown in Fig 6, there were no changes in the protein levels of the CDK inhibitors p21 and p27 in C2C12 myoblasts treated with As_2_O_3_ (0.25–1 μM) for 48 h. Moreover, G1 progression and the G1/S transition are regulated by cyclin D1, which activates CDK 4 and CDK6, and cyclin E, which activates CDK2. Treatment of C2C12 myoblasts with low-concentrations of As_2_O_3_ for 48 h decreased the protein levels of CDK2, CDK4, cyclin E, and cyclin D1 in a dose-dependent manner. Moreover, the cyclin B1 and CDC-2 proteins are related to the progression of G2 phase [27,28]. We further found that As_2_O_3_ treatment down-regulated cyclin B1, but not cdc 2, protein expressions (Fig 6). These results indicate that low-concentrations of As_2_O_3_ inhibit the CDK and cyclin expression, which are involved in the transition from G1/to S and G2 to M phase.

### Effect of low-concentration As_2_O_3_ on the phosphorylation of Akt

**Fig 7:**
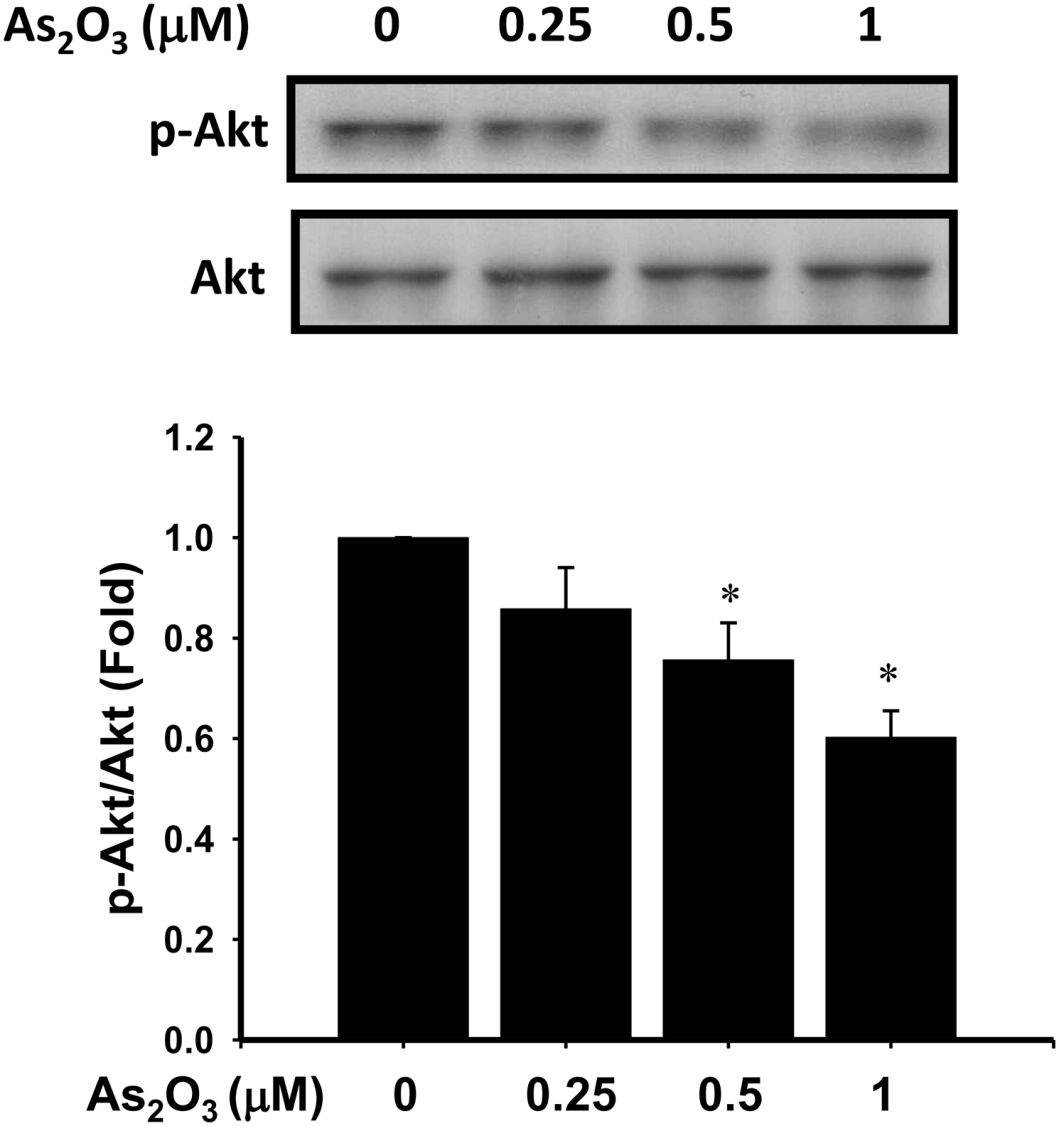
As_2_O_3_ decreases the phosphorylation of Akt in myoblasts. C2C12 myoblasts were harvested at 48 h after incubation with As_2_O_3_ (0.25–1 μM). The phosphorylation of Akt and total Akt protein were determined by Western blotting. Protein expression was quantified by densitometry. Data are presented as means ± SEM of three independent experiments. *P < 0.05 vs control.

**Fig 8:**
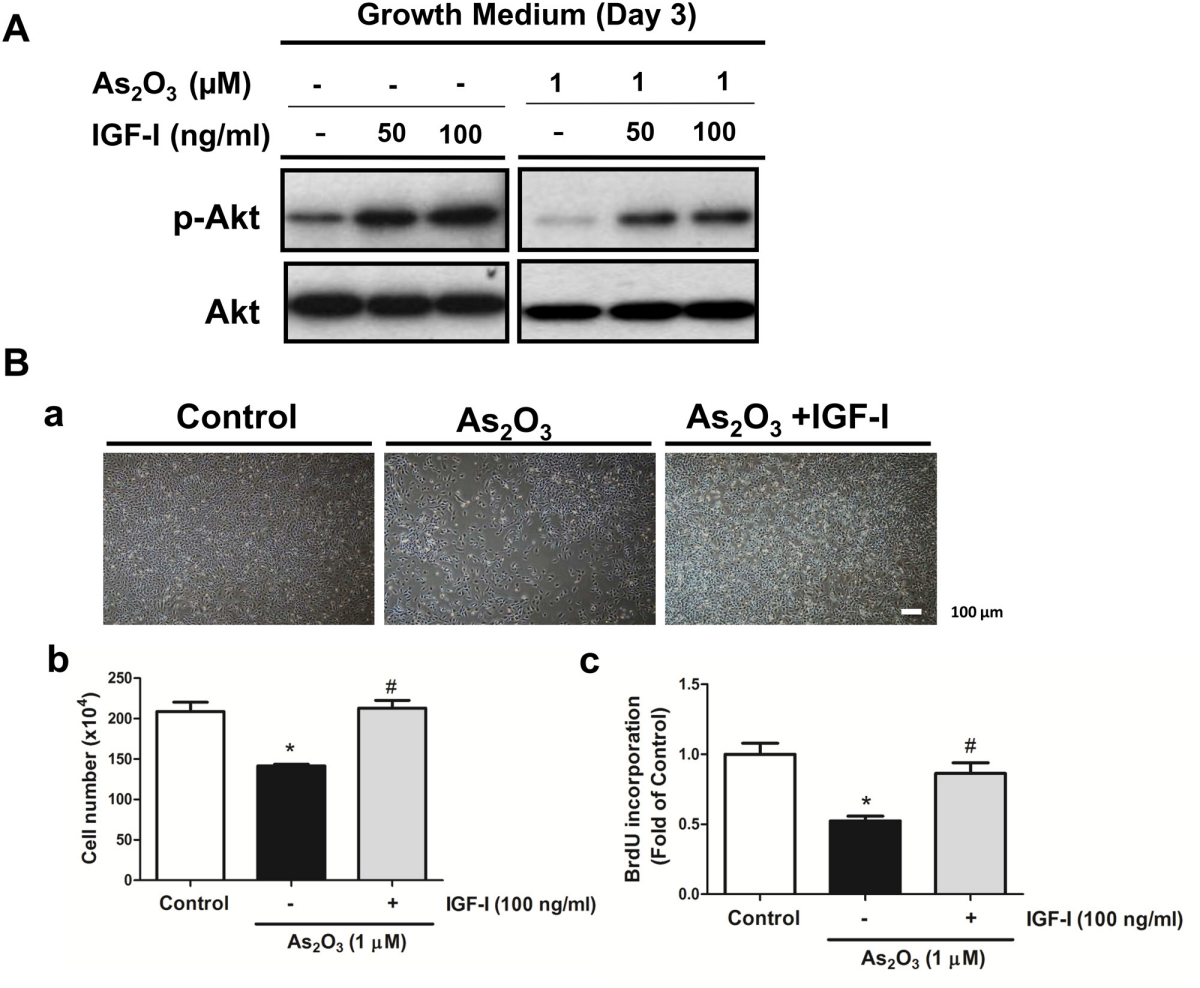
Inhibitory effects of As_2_O_3_ on Akt phosphorylation and cell proliferation are reversed by insulin-like growth factor 1 (IGF-I). C2C12 myoblasts were pretreated with IGF-I (50 and 100 ng/ml) for 1 h and then incubated with 1 μM As_2_O_3_ for 72 h. (A) The phosphorylation of Akt and total Akt protein were determined by Western blotting. (B) Cell morphology was observed under an inverted phase contrast microscope (a). Cells were counted after staining with 0.4% trypan blue (b) and bromodeoxyuridine (BrdU) incorporation assay (c). All data are presented as means ± SEM for at least three independent experiments. Scale bar = 100 μm. *P < 0.05 vs control; ^#^ *P* < 0.05 vs As_2_O_3_.

Akt (also known as protein kinase B) signaling pathway coordinates or synergistically promotes cell growth and progression throughout the cell cycle [29]. We investigated whether or not the As_2_O_3_-induced myoblast proliferation inhibition was associated with Akt signaling pathway. As shown in Fig 7, As_2_O_3_ (2.5–1 μM) suppressed the phosphorylation of Akt (at Ser473) in a dose-dependent manner, whereas total form of Akt protein expression was not altered. Moreover, the insulin-like growth factor-1(IGF-1)/Akt signaling pathway is involved in the positive regulation of skeletal muscle mass [30]. IGF-1 (50 and 100 ng/ml) markedly increased the Akt phosphorylation in myoblasts and reversed the Akt phosphorylation inhibition by As_2_O_3_ (1 μM) (Fig 8A). IGF-1 also significantly reversed the inhibition of myoblast cell proliferation by As_2_O_3_ (1 μM) (Fig 8B).

## Discussion

As_2_O_3_ is an active agent for leukemia (APL) therapy [5]. As_2_O_3_ has also been considered to be a novel therapeutic agent for lymphoproliferative and autoimmune syndromes [31]. The plasma arsenic levels performed a peak level of 5.54 μM to 7.30 μM in patients for which As_2_O_3_ was administered intravenously at the dose of 10 mg/d for APL treatment [32]. As_2_O_3_ was reported to induce acute promyelocytic leukemia cell differentiation at lower concentrations (0.1–0.5 μM), but induce cell apoptosis at higher concentrations (0.5–2 μM) [33]. It has been shown that arsenic level of > 0.64 mg/L in drinking water is associated with an increase in liver cancer mortality in both sexes [34]. In addition, high concentrations of As_2_O_3_ (30, 60, and 90 μM) for various periods (24, 48, and 72 h) caused apoptosis in primary cardiomyocytes [35]. As_2_O_3_ (5–7 μM) also increased oxidative stress, mitochondrial dysfunctions, or apoptosis in H9c2 cardiomyoblasts [36–38]. On the other hand, our previous studies showed that C2C12 myoblasts and primary mouse and human myoblasts cultured in differentiation media with As_2_O_3_ (0.1–0.5 μM) for 4 days significantly inhibited the myoblast differentiation [22]. Moreover, C2C12 myoblasts underwent apoptosis in response to As_2_O_3_ (3–10 μM) for 24 h of treatment [24]. In the present study, the myoblast growth (proliferation) was decreased by As_2_O_3_ at submicromolar concentrations (0.25–1 μM) after 72 h of exposure. These results indicated that As_2_O_3_ at the concentrations of 0.5–1 μM may not only affect the myoblast differentiation but also inhibit myoblast cell growth without apoptosis induction. The findings also suggest that exposure to As_2_O_3_ at doses relevant to human exposure may alter the myoblast proliferation and may interfere with the skeletal muscle cell development/growth.

Muscle growth, maintenance, and repair of injured muscle fibers require myogenesis [39]. Decreased proliferation of myoblasts could reduce the number of muscle fibers. C2C12 myoblasts are a good *in vitro* model for myoblast proliferation and differentiation and are easily reproducible in cell cultures [40,41]. We found that treatment with 0.25–1 μM As_2_O_3_ resulted in the inhibition of C2C12 myoblast growth in a dose-dependent manner. Moreover, PCNA is an auxiliary protein of DNA polymerase δ, the level of which correlates with DNA synthesis during the cell cycle. The PCNA level is maximal during the S-phase of the cell cycle [42]. PCNA is also a marker for evaluating the proliferation activity of cells [25]. Our data showed that low-concentration As_2_O_3_ induced decreases in nuclear PCNA protein expression and BrdU incorporation in the myoblasts, but did not induce cell apoptosis. We also found that arsenic contents are increased in myoblasts after the treatment with low-concentration As_2_O_3_, indicating that As_2_O_3_ enters into myoblasts. Unexpectedly, As_2_O_3_ (0.5 and 1 μM) did not affect the generation of ROS in myoblasts. These findings indicate that As_2_O_3_ at submicromolar concentrations inhibits skeletal myoblast proliferation without cytotoxicity and its mechanism of action does not depend on ROS.

The cell cycle is regulated by complexes of cyclins and cyclin-dependent kinases (CDKs), whose formation and activation promote the cell cycle progression [43]. The main targets for this regulation are the cyclin-CDK complexes, cyclin-D1/CDK4 and cyclin-E/CDK2 [43,44]. These complexes control the G1 to S transition through phosphorylation and inactivation of the retinoblastoma protein and PCNA expression [44], while Cdc2 protein and cyclin B1 regulate the progression of G2/M phase [27]. As_2_O_3_ (1–2 μM) inhibits the proliferation of human endothelial cells by preventing the cell cycle progression from G1 to S and by causing G2/M phase arrest of the cell cycle [45]. Yih and Lee (2000) also reported that arsenic (5 μM) induced G2/M arrest with no apoptosis in human fibroblasts [12]. Renal cell carcinoma A498 cells treated with 2.5 μM As_2_O_3_ for 72 h resulted in a down-regulation of cyclin D1 [46]. In addition, As_2_O_3_ down-regulates cyclin D1 transcription via a reduction of Sp1 transcription factor in gallbladder carcinoma cells [47]. In the present study, the results of myoblast cell cycle analysis showed that 0.5 and 1 μM As_2_O_3_ induced G1 phase cell cycle arrest. These cells therefore could not make the transition to the S-phase of the cell cycle, altering the progression of G2 to M phase. Moreover, 0.5 and 1 μM As_2_O_3_ significantly decreased the cyclin D1 and CDK4 protein levels in a dose-dependent manner. This indicates that arsenic-mediated G1 cell cycle arrest and down-regulation of cyclin E protein in myoblasts are related to suppression of cyclin D1 and CDK4. Low-concentration As_2_O_3_ also led to a marked dose-dependent decrease in cyclin B1 expression, suggesting As_2_O_3_ induced G2/M phase arrest. On the other hand, the up-regulation of CDK inhibitors can inhibit the cyclin-CDK complexes and causes cell cycle arrest [48]. However, the present work showed that neither p21 nor p27 protein expressions are affected by the treatment of low-concentration As_2_O_3_ in myoblasts. Thus, it is likely that the CDK inhibitors p21 and p27 may not play a significant role in the cell cycle arrest in As_2_O_3_-treated myoblasts.

An accumulation of evidence supports a crucial role for Akt activation in regulating muscle cell survival, proliferation, and differentiation [22,24,49–51]. The proliferation of C2C12 myoblasts induced by insulin depends on Akt activity [52]. Using a specific RNA interference, the Akt1 isoform is required for C2 myoblast cell proliferation, while the Akt2 isoform plays a negative effect on cell cycle progression [53]. Over-expression of constitutive Akt (myrAkt) activity extended the half-life of cyclin D1 protein levels, whereas treatment with wortmannin (a PI3K inhibitor) accelerated cyclin D1 degradation [54]. Yang et al. (2007) reported that the PI3K/Akt/GSK-3β signaling cascade participates in myostatin-stimulated cyclin D1 degradation and C2C12 myoblast proliferation inhibition [51]. Moreover, IGF-1-activated Akt signaling increases muscle mass through the induction of protein synthesis [30]. IGF-1 has also markedly increased the cyclin D1 expression in proliferating C2C12 myoblasts [55]. In the present study, we observed that As_2_O_3_ at submicromolar concentrations inhibited the cell proliferation and phosphorylation of Akt and protein expression of cyclin D1 in C2C12 myoblasts in a dose-dependent manner. IGF-1 significantly reversed the inhibitory effect of As_2_O_3_ on Akt phosphorylation and cell proliferation in myoblasts. These results indicate that the decreased phosphorylation of Akt may be partially responsible for the suppression of cyclin D1 protein levels and proliferation of myoblasts exposed to low-concentration As_2_O_3_.

In conclusion, the results of this study provide evidence that As_2_O_3_ at submicromolar concentrations inhibits skeletal myoblast proliferation and induces cell cycle arrest. ROS are most likely not involved in the inhibitory effect of As_2_O_3_ on myoblast proliferation. Low-concentration As_2_O_3_ also suppressed CDK and cyclin expressions, which may be, at least in part, associated with the inhibition of Akt signaling. However, the more detailed mechanisms still need to be clarified in the future. Based on these results we suggest that As_2_O_3_ interferes with myoblast growth and may be an environmental risk factor for skeletal muscle cell development and growth.

## References

[1] Kumagai Y, Sumi D. Arsenic: signal transduction, transcription factor, and biotransformation involved in cellular response and toxicity. Annu Rev Pharmacol Toxicol. 2007; 47: 243–262. 17002598 10.1146/annurev.pharmtox.47.120505.105144

[2] Tapio S, Grosche B. Arsenic in the aetiology of cancer. Mutat Res. 2006; 612: 215–246. 16574468 10.1016/j.mrrev.2006.02.001

[3] Efferth T, Li PC, Konkimalla VS, Kaina B. From traditional Chinese medicine to rational cancer therapy. Trends Mol Med. 2007; 13: 353–361. 17644431 10.1016/j.molmed.2007.07.001

[4] Miller WH Jr, Schipper HM, Lee JS, Singer J, Waxman S. Mechanisms of action of arsenic trioxide. Cancer Res. 2002; 62: 3893–3903. 12124315

[5] Dilda PJ, Hogg PJ. Arsenical-based cancer drugs. Cancer Treat Rev. 2007; 33: 542–564. 17624680 10.1016/j.ctrv.2007.05.001

[6] Smith AH, Marshall G, Yuan Y, Ferreccio C, Liaw J, von Ehrenstein O, et al Increased mortality from lung cancer and bronchiectasis in young adults after exposure to arsenic in utero and in early childhood. Environ Health Perspect. 2006; 114: 1293–1296. 16882542 10.1289/ehp.8832 PMC1551995

[7] Waalkes MP, Liu J, Diwan BA. Transplacental arsenic carcinogenesis in mice. Toxicol Appl Pharmacol. 2007; 222: 271–280. 17306315 10.1016/j.taap.2006.12.034 PMC1995036

[8] Watcharasit P, Visitnonthachai D, Suntararuks S, Thiantanawat A, Satayavivad J. Low arsenite concentrations induce cell proliferation via activation of VEGF signaling in human neuroblastoma SH-SY5Y cells. Environ Toxicol Pharmacol. 2012; 33: 53–59. 10.1016/j.etap.2011.10.005 22120617

[9] Kligerman AD, Tennant AH. Insights into the carcinogenic mode of action of arsenic. Toxicol Appl Pharmacol. 2007; 222: 281–288. 17118416 10.1016/j.taap.2006.10.006

[10] Zhang W, Ohnishi K, Shigeno K, Fujisawa S, Naito K, Nakamura S, et al The induction of apoptosis and cell cycle arrest by arsenic trioxide in lymphoid neoplasms. Leukemia. 1998; 12: 1383–1391. 9737686 10.1038/sj.leu.2401112

[11] Seol JG, Park WH, Kim ES, Jung CW, Hyun JM, Kim BK, et al Effect of arsenic trioxide on cell cycle arrest in head and neck cancer cell line PCI-1. Biochem Biophys Res Commun. 1999; 265: 400–404. 10558879 10.1006/bbrc.1999.1697

[12] Yih LH, Lee TC. Arsenite induces p53 accumulation through an ATM-dependent pathway in human fibroblasts. Cancer Res. 2000; 60: 6346–6352. 11103796

[13] Chen F, Zhang Z, Bower J, Lu Y, Leonard SS, Ding M, et al Arsenite-induced Cdc25C degradation is through the KEN-box and ubiquitin-proteasome pathway. Proc Natl Acad Sci USA. 2002; 99: 1990–1995. 11842186 10.1073/pnas.032428899 PMC122307

[14] Li X, Ding X, Adrian TE. Arsenic trioxide causes redistribution of cell cycle, caspase activation, and GADD expression in human colonic, breast, and pancreatic cancer cells. Cancer Invest. 2004; 22:389–400. 15493360 10.1081/cnv-200029068

[15] Mauro A. Satellite cell of skeletal muscle fibers. J Biophys Biochem Cytol. 1961; 9: 493–495. 13768451 10.1083/jcb.9.2.493 PMC2225012

[16] Mitchell PO, Pavlath GK. A muscle precursor cell-dependent pathway contributes to muscle growth after atrophy. Am J Physiol Cell Physiol. 2001; 281: C1706–C1715. 11600435 10.1152/ajpcell.2001.281.5.C1706

[17] Charge SB, Rudnicki MA. Cellular and molecular regulation of muscle regeneration. Physiol Rev. 2004; 84: 209–238. 14715915 10.1152/physrev.00019.2003

[18] Allen DL, Monke SR, Talmadge RJ, Roy RR, Edgerton VR. Plasticity of myonuclear number in hypertrophied and atrophied mammalian skeletal muscle fibers. J Appl Physiol. 1995; 78: 1969–1976. 7649936 10.1152/jappl.1995.78.5.1969

[19] Brameld JM, Mostyn A, Dandrea J, Stephenson TJ, Dawson JM, Buttery PJ, et al Maternal nutrition alters the expression of insulin-like growth factors in fetal sheep liver and skeletal muscle. J Endocrinol. 2000; 167: 429–437. 11115769 10.1677/joe.0.1670429

[20] Fahey AJ, Brameld JM, Parr T, Buttery PJ. The effect of maternal undernutrition before muscle differentiation on the muscle fiber development of the newborn lamb. J Anim Sci. 2005; 83: 2564–2571. 16230653 10.2527/2005.83112564x

[21] Steffens AA, Hong GM, Bain LJ. Sodium arsenite delays the differentiation of C2C12 mouse myoblast cells and alters methylation patterns on the transcription factor myogenin. Toxicol Appl Pharmacol. 2011; 250: 154–161. 10.1016/j.taap.2010.10.006 20965206 PMC3014457

[22] Yen YP, Tsai KS, Chen YW, Huang CF, Yang RS, Liu SH. Arsenic inhibits myogenic differentiation and muscle regeneration. Environ Health Perspect. 2010; 118: 949–956. 10.1289/ehp.0901525 20299303 PMC2920914

[23] Wu CT, Lu TY, Chan DC, Tsai KS, Yang RS, Liu SH. Effects of arsenic on osteoblast differentiation in vitro and on bone mineral density and microstructure in rats. Environ Health Perspect. 2014; 122: 559–565. 10.1289/ehp.1307832 24531206 PMC4050517

[24] Yen YP, Tsai KS, Chen YW, Huang CF, Yang RS, Liu SH. Arsenic induces apoptosis in myoblasts through a reactive oxygen species-induced endoplasmic reticulum stress and mitochondrial dysfunction pathway. Arch Toxicol. 2012; 86: 923–933. 10.1007/s00204-012-0864-9 22622864

[25] Schabort EJ, van der Merwe M, Loos B, Moore FP, Niesler CU. TGF-beta's delay skeletal muscle progenitor cell differentiation in an isoform-independent manner. Exp Cell Res. 2009; 315: 373–384. 10.1016/j.yexcr.2008.10.037 19038250

[26] Chen YC, Lin-Shiau SY, Lin JK. Involvement of reactive oxygen species and caspase 3 activation in arsenite-induced apoptosis. J Cell Physiol. 1998; 177: 324–333. 9766529 10.1002/(SICI)1097-4652(199811)177:2<324::AID-JCP14>3.0.CO;2-9

[27] Stark GR, Taylor WR. Control of the G2/M transition. Mol Biotechnol. 2006; 32: 227–248. 16632889 10.1385/MB:32:3:227

[28] Tsai LH, Harlow E, Meyerson M. Isolation of the human cdk2 gene that encodes the cyclin A- and adenovirus E1A-associated p33 kinase. Nature. 1991; 353: 174–177. 1653904 10.1038/353174a0

[29] Worster DT, Schmelzle T, Solimini NL, Lightcap ES, Millard B, Mills GB, et al Akt and ERK control the proliferative response of mammary epithelial cells to the growth factors IGF-1 and EGF through the cell cycle inhibitor p57^kip2^ . Sci Signal. 2012; 5: ra19 10.1126/scisignal.2001986 22394561 PMC4174537

[30] Egerman MA, Glass DJ. Signaling pathways controlling skeletal muscle mass. Crit Rev Biochem Mol Biol. 2014; 49: 59–68. 10.3109/10409238.2013.857291 24237131 PMC3913083

[31] Bobé P, Bonardelle D, Benihoud K, Opolon P, Chelbi-Alix MK. Arsenic trioxide: A promising novel therapeutic agent for lymphoproliferative and autoimmune syndromes in MRL/lpr mice. Blood. 2006; 108: 3967–3975. 16926289 10.1182/blood-2006-04-020610

[32] Shen ZX, Chen GQ, Ni JH, Li XS, Xiong SM, Qiu QY, et al Use of arsenic trioxide (As2O3) in the treatment of acute promyelocytic leukemia (APL): II. Clinical efficacy and pharmacokinetics in relapsed patients. Blood. 1997; 89: 3354–3360. 9129042

[33] Chen GQ, Shi XG, Tang W, Xiong SM, Zhu J, Cai X, et al Use of arsenic trioxide (As2O3) in the treatment of acute promyelocytic leukemia (APL): I. As2O3 exerts dose-dependent dual effects on APL cells. Blood. 1997; 89: 3345–3353. 9129041

[34] Lin HJ, Sung TI, Chen CY, Guo HR. Arsenic levels in drinking water and mortality of liver cancer in Taiwan. J Hazard Mater. 2013; 262: 1132–1138. 10.1016/j.jhazmat.2012.12.049 23352725

[35] Raghu KG, Cherian OL. Characterization of cytotoxicity induced by arsenic trioxide (a potent anti-APL drug) in rat cardiac myocytes. J Trace Elem Med Biol. 2009; 23: 61–68. 10.1016/j.jtemb.2008.10.001 19203718

[36] Vineetha VP, Prathapan A, Soumya RS, Raghu KG. Arsenic trioxide toxicity in H9c2 myoblasts—damage to cell organelles and possible amelioration with *Boerhavia diffusa* . Cardiovasc Toxicol. 2013; 13: 123–137. 10.1007/s12012-012-9191-x 23161055

[37] Vineetha VP, Girija S, Soumya RS, Raghu KG. Polyphenol-rich apple (*Malus domestica L*.) peel extract attenuates arsenic trioxide induced cardiotoxicity in H9c2 cells via its antioxidant activity. Food Funct. 2014; 5: 502–511. 10.1039/c3fo60470e 24441683

[38] Vineetha VP, Soumya RS, Raghu KG. Phloretin ameliorates arsenic trioxide induced mitochondrial dysfunction in H9c2 cardiomyoblasts mediated via alterations in membrane permeability and ETC complexes. Eur J Pharmacol. 2015; 754: 162–172. 10.1016/j.ejphar.2015.02.036 25746422

[39] Parker MH, Seale P, Rudnicki MA. Looking back to the embryo: defining transcriptional networks in adult myogenesis. Nat Rev Genet. 2003; 4: 497–507. 12838342 10.1038/nrg1109

[40] Joulia D, Bernardi H, Garandel V, Rabenoelina F, Vernus B, Cabello G. Mechanisms involved in the inhibition of myoblast proliferation and differentiation by myostatin. Exp Cell Res. 2003; 286: 263–275. 12749855 10.1016/s0014-4827(03)00074-0

[41] Yaffe D, Saxel O. Serial passaging and differentiation of myogenic cells isolated from dystrophic mouse muscle. Nature. 1977; 270: 725–727. 563524 10.1038/270725a0

[42] Hawke TJ, Garry DJ. Myogenic satellite cells: physiology to molecular biology. J Appl Physiol. 2001; 91: 534–551. 11457764 10.1152/jappl.2001.91.2.534

[43] Weinberg RA. The retinoblastoma protein and cell cycle control. Cell. 1995; 81: 323–330. 7736585 10.1016/0092-8674(95)90385-2

[44] Massague J. G1 cell-cycle control and cancer. Nature. 2004; 432: 298–306. 15549091 10.1038/nature03094

[45] Woo SH, Park MJ, An S, Lee HC, Jin HO, Lee SJ, et al Diarsenic and tetraarsenic oxide inhibit cell cycle progression and bFGF- and VEGF-induced proliferation of human endothelial cells. J Cell Biochem. 2005; 95: 120–130. 15723287 10.1002/jcb.20329

[46] Hyun Park W, Hee Cho Y, Won Jung C, Oh Park J, Kim K, Hyuck Im Y, et al Arsenic trioxide inhibits the growth of A498 renal cell carcinoma cells via cell cycle arrest or apoptosis. Biochem Biophys Res Commun. 2003; 300: 230–235. 12480548 10.1016/s0006-291x(02)02831-0

[47] Ai Z, Lu W, Ton S, Liu H, Sou T, Shen Z, et al Arsenic trioxide-mediated growth inhibition in gallbladder carcinoma cells via down-regulation of Cyclin D1 transcription mediated by Sp1 transcription factor. Biochem Biophys Res Commun. 2007; 360: 684–689. 17617380 10.1016/j.bbrc.2007.06.123

[48] Sherr CJ, Roberts JM. Inhibitors of mammalian G1 cyclin-dependent kinases. Genes Dev. 1995; 9: 1149–1163. 7758941 10.1101/gad.9.10.1149

[49] Ji M, Zhang Q, Ye J, Wang X, Yang W, Zhu D. Myostatin induces p300 degradation to silence cyclin D1 expression through the PI3K/PTEN/Akt pathway. Cell Signal. 2008; 20: 1452–1458. 10.1016/j.cellsig.2008.03.013 18472397

[50] Kaliman P, Vinals F, Testar X, Palacin M, Zorzano A. Phosphatidylinositol 3-kinase inhibitors block differentiation of skeletal muscle cells. J Biol Chem. 1996; 271: 19146–19151. 8702591 10.1074/jbc.271.32.19146

[51] Yang W, Zhang Y, Li Y, Wu Z, Zhu D. Myostatin induces cyclin D1 degradation to cause cell cycle arrest through a phosphatidylinositol 3-kinase/AKT/GSK-3β pathway and is antagonized by insulin-like growth factor 1. J Biol Chem. 2007; 282: 3799–3808. 17130121 10.1074/jbc.M610185200

[52] Conejo R, Lorenzo M. Insulin signaling leading to proliferation, survival, and membrane ruffling in C2C12 myoblasts. J Cell Physiol. 2001; 187: 96–108. 11241354 10.1002/1097-4652(2001)9999:9999<::AID-JCP1058>3.0.CO;2-V

[53] Heron-Milhavet L, Franckhauser C, Rana V, Berthenet C, Fisher D, Hemmings BA, et al Only Akt1 is required for proliferation, while Akt2 promotes cell cycle exit through p21 binding. Mol Cell Biol. 2006; 26: 8267–8280. 16982699 10.1128/MCB.00201-06 PMC1636765

[54] Diehl JA, Cheng M, Roussel MF, Sherr CJ. Glycogen synthase kinase-3beta regulates cyclin D1 proteolysis and subcellular localization. Genes Dev. 1998; 12: 3499–3511. 9832503 10.1101/gad.12.22.3499 PMC317244

[55] Grabiec K, Gajewska M, Milewska M, Błaszczyk M, Grzelkowska-Kowalczyk K. The influence of high glucose and high insulin on mechanisms controlling cell cycle progression and arrest in mouse C2C12 myoblasts: the comparison with IGF-I effect. J Endocrinol Invest. 2014; 37: 233–245. 10.1007/s40618-013-0007-z 24615360 PMC3949044

